# Impact of plain packaging of tobacco products on smoking in adults and children: an elicitation of international experts’ estimates

**DOI:** 10.1186/1471-2458-13-18

**Published:** 2013-01-09

**Authors:** Rachel Pechey, David Spiegelhalter, Theresa M Marteau

**Affiliations:** 1 Behaviour and Health Research Unit, Institute of Public Health, Cambridge CB2 0SR, UK; 2 Statistical Laboratory, Centre for Mathematical Sciences, Cambridge CB3 0WB, UK

**Keywords:** Tobacco, Plain packaging, Expert elicitation

## Abstract

**Background:**

Governments sometimes face important decisions in the absence of direct evidence. In these cases, expert elicitation methods can be used to quantify uncertainty. We report the results of an expert elicitation study regarding the likely impact on smoking rates in adults and children of plain packaging of tobacco products.

**Methods:**

Thirty-three tobacco control experts were recruited from the UK (n = 14), Australasia (n = 12) and North America (n = 7). Experts’ estimates were individually elicited via telephone interviews, and then linearly pooled. Elicited estimates consisted of (1) the most likely, (2) the highest possible, and (3) the lowest possible value for the percentage of (a) adult smokers and (b) children trying smoking, two years after the introduction of plain packaging (all other things being constant) in a target country in the expert’s region of residence.

**Results:**

The median estimate for the impact on adult smoking prevalence was a 1 percentage point decline (99% range 2.25 to 0), and for the percentage of children trying smoking was a 3 percentage point decline (99% range 6.1 to 0), the latter estimated impact being larger than the former (P < 0.001, sign test). There were no differences in either estimate by region (I^2^: Adults: 0; Children: 0) but there was considerable variability between experts’ estimates within regions (I^2^: Adults: 0.91; Children: 0.89).

**Conclusions:**

In the absence of direct evidence for the impact of introducing plain packaging on smoking rates in adults and children, this study shows that tobacco control experts felt the most likely outcomes would be a reduction in smoking prevalence in adults, and a greater reduction in the numbers of children trying smoking, although there was substantial variability in the estimated size of these impacts. No experts judged an increase in smoking as a likely outcome.

## Background

Governments and others in authority sometimes face important decisions in the absence of direct quantifiable evidence. Expert elicitation methods have been developed to quantify uncertainty in such contexts including estimating risks of volcanic eruptions [1], climate change [2] and effect sizes in clinical trials [3]. We report a study using this method to quantify uncertainty regarding the likely impact on smoking rates of plain packaging of tobacco products.

The WHO Framework Convention on Tobacco Control includes packaging as one of the core non-price demand reduction measures, whereby “packaging and labelling do not promote a tobacco product by any means that are false, misleading, deceptive or likely to create an erroneous impression about its characteristics, health effects, hazards or emissions” [4]. Having overcome a high court challenge, Australia is the first to sell all tobacco products in plain packaging (i.e. without brand imagery or promotional text, and using standardised formatting) [5,6], while the UK government is conducting a public consultation on the possible introduction of such a policy [7]. As yet, however, this measure has only just been implemented by the first country to adopt this policy, so the evidence available to anticipate the impact of such a policy is inevitably indirect. Such evidence includes experimental and observational studies of the impact on attitudes and behaviour of various types of cigarette packaging [8-12].

Two systematic reviews of this indirect evidence have described three ways in which plain packaging may reduce smoking rates, particularly amongst children and young adults: first, by reducing the appeal of packs; second, by increasing the salience of health warnings; and third, by standardising pack colour, thus avoiding perceptions of this as an indicator of product harmfulness [13,14].

As this evidence is necessarily indirect, its relevance has been questioned (for example, the strength of the relationship between thinking about quitting, a common outcome measure in this set of evidence, and actual quitting), and doubts raised too as to the strength of anti-smoking campaigners’ beliefs about the likelihood that plain packaging will reduce rates of smoking [15].

At least two recent reports suggest that plain packaging could increase smoking: first, by reducing product differentiation, leading to smokers buying cheaper brands; and second, by increasing smuggling and counterfeit products, thereby increasing the availability of cheaper cigarettes [15,16]. The assumptions underlying these predictions have, however, been contested [17]. The first of these reports was funded by an organisation that receives some funding from the tobacco industry [15], the second by the tobacco industry [16], while the third is funded by a campaigning public health charity, which receives funding from health-related charities as well as the UK government Department of Health [17].

This study aims to elicit estimates of international tobacco control experts on the likely impact of plain packaging of tobacco products on smoking prevalence in adults and the percentage of children trying smoking. The impact on children is of particular importance given that the majority of smokers first try smoking in adolescence, with nicotine dependence developing rapidly thereafter, even before the user becomes a regular (weekly) smoker [18].

## Method

### Sample

The sample comprised internationally-renowned experts on tobacco control policies, recruited from three geographical regions where plain packaging policies are in the process of being implemented or are under active consideration or discussion (Australasia, UK, North America). Participants were identified from editorial lists for relevant journals (Addiction; Tobacco Control; Nicotine and Tobacco Research), and leadership positions in the Society for Research on Nicotine and Tobacco. Experts met Hora and van Winterfeldt’s [19] first four requirements for participation in expert elicitation, that is: (a) tangible evidence of expertise (as evidenced by publications), (b) reputation (as indicated by peer-nomination), and (c) availability and willingness to participate, (d) understanding of the general problem area. To address the latter two requirements suggested by Hora and van Winterfeldt (impartiality and lack of an economic or personal stake in potential findings), we include a description of the participants’ competing interests for transparency. Forty-five experts (15 from each region) were invited to participate, of whom 33 accepted (UK: 14/15; Australasia: 12/15; and North America: 7/15). Previous studies have found 5–6 participants per group to be sufficient for group estimates [20,21].

The study received ethical approval from the Psychology Research Ethics Committee of the University of Cambridge [Ref. 2011.77]. Participants gave informed consent before taking part.

### Procedure

A semi-structured telephone interview was used to elicit subjective judgments for the impact of plain packaging on (a) the prevalence of smoking in adults and (b) the percentage of children trying smoking. The script was developed by the authors from those used in similar studies [22-24]. Prior to interview participants were sent a copy of a recent systematic review on the impact of plain packaging of tobacco products [14] to ensure that all participants had the same summary of the most recent evidence relating to plain packaging. This did not provide numerical estimates of the likely impact of plain packaging policies on the two outcomes of interest in this study. During the interview, the interviewer provided the prevalence rates for the two outcomes of interest and asked participants to estimate the expected values of these two years after the introduction of plain packaging in their region, and the lowest and highest likely values, holding all other relevant factors constant (e.g. with current controls regarding the sale of tobacco still being in force, and the price and current prevalence levels [25-29] being stable over the two year period). Subsidiary questions were used to explore the range of plausible values provided, to ensure that experts felt they would be extremely surprised if the actual values fell outside the range they had provided (‘extremely’ was described as a 1% chance), given the tendency of individuals to provide too narrow a range in these types of study [30]. Finally, participants were asked to outline the reasoning behind the estimates they provided.

### Analysis

Expert estimates were linearly pooled to obtain medians of best estimates, lower, and upper points, which are presented as summary statistics, to represent the opinion of an ‘average expert’. Impact, measured as absolute percentage change, is displayed on forest plots to distinguish within-person uncertainty from between-subject variability. Given the observed between-expert variation in median change, with 32 observations the overall median percentage change could be estimated with approximate standard error 0.16 for adults and 0.32 for children.

Non-parametric tests (Kruskal-Wallis, sign) were used for differences in best-estimates between regions and adult/child estimates. Finally, I^2^, the proportion of variability due to heterogeneity, is used in an informal manner here, as the estimates generated in the current study are not based on independent samples, but subjective opinions that we can expect to be correlated. The ‘standard errors’ that are used in the calculation of I^2^ were taken as the range provided by each expert divided by 5.2, as if the range were a 99% normal interval.

## Results

### Quantitative

**Figure 1:**
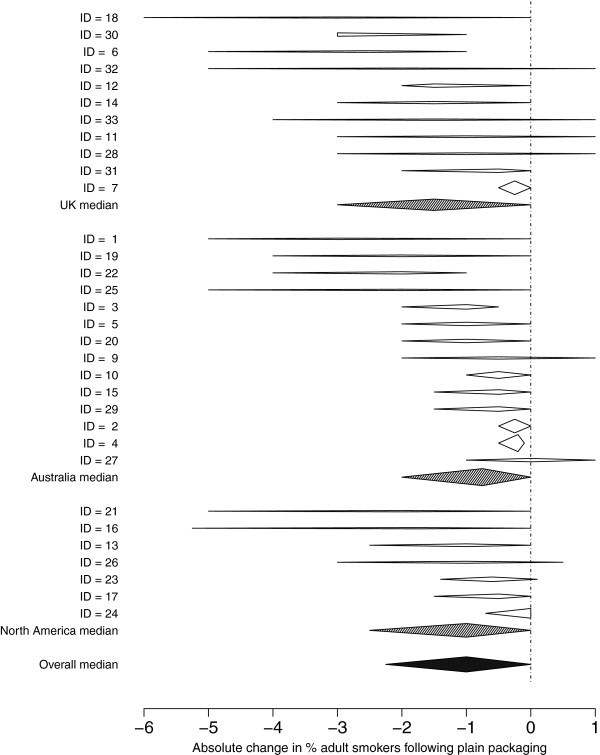
**Forest plot of estimates of absolute change in the prevalence of adult smokers two years after the introduction of plain packaging (holding other factors constant).** [Prevalence rates provided to experts were: Britain: 21%; Australia: 18%; Canada: 17.5%].

**Figure 2:**
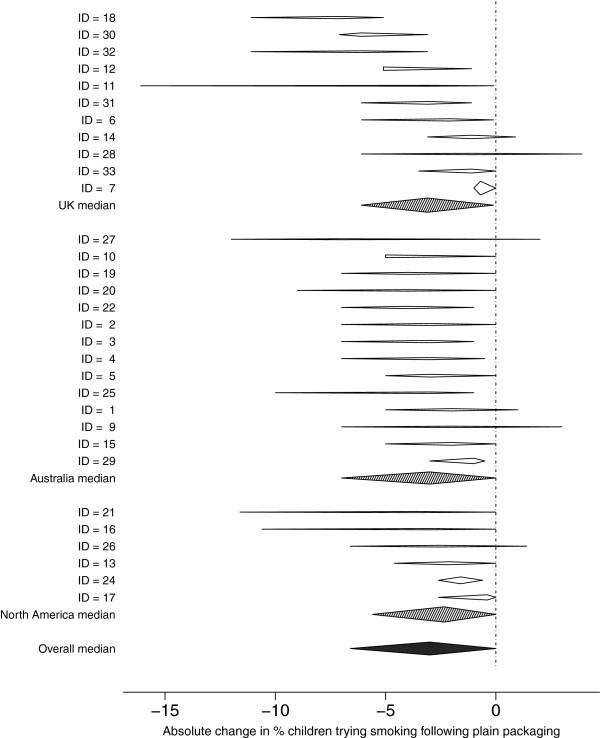
**Forest plot of estimates of absolute change in the prevalence of children trying smoking two years after the introduction of plain packaging (holding other factors constant). **[Prevalence rates provided to experts were: Britain: 27%; Australia: 21.1%; Canada: 21.6%].

Estimates of the prevalence of smoking following plain packaging were provided by 14/14 UK, 11/12 Australasian, and 7/7 North American participants. Similar numbers provided estimates for the percentage of children trying smoking with the exception of North American participants, of whom 6/7 provided estimates. These estimates were subtracted from the region-specific baseline rates to produce absolute changes expressed as percentage points, as shown in Figures 1 and 2.

The overall median estimate for the absolute change in the prevalence of adults smoking two years after the introduction of plain packaging was −1% (between-expert range −3% to 0%). The median estimates for the lowest and highest values were −2.25% (between-expert range −6% to −0.5%) and 0% (between-expert range −1% to 1%).

The overall median estimate for the absolute change in the percentage of children trying smoking two years after the introduction of plain packaging was −3% (between-expert range −7.1% to −0.4%). The median estimates for the lowest and highest values were −6.1% (between-expert range −16.1% to −1%) and 0% (between-expert range −5.1% to 3.9%).

The majority (26/31) of experts had a larger ‘best estimate’ of the absolute effect on children than adults (P < 0.001, sign test).

There was no evidence for systematic difference between regions for estimates of either the prevalence of smoking in adults or for the percentage of children trying smoking (Adults: Kruskal-Wallis chi-squared = 3.06, df = 2, p-value = 0.22; Children: Kruskal-Wallis chi-squared = 1.59, df = 2, p-value = 0.45). The I^2^ between regions for both adults and children was 0. However, there was strong heterogeneity within regions (I^2^ within regions (adults): 0.91; I^2^ within regions (children): 0.89).

### Qualitative

We present examples of the reasoning provided by participants when generating these estimates below. Most experts (n = 20) explicitly stated that they would expect a larger impact of plain packaging on the numbers of children smoking, expecting younger people to be more affected by less appealing packs, less brand identification, and changes in social norms around smoking. In contrast, an impact on cessation-related behaviours was less frequently mentioned (n = 9), reflecting a view that plain packaging would have little impact on more-established, heavily addicted smokers. Many participants (n = 14) also felt that the two year time frame for which estimates were requested did not allow for the full impact of plain packaging to be seen in prevalence rates. Additionally, several participants (n = 12) noted that in reality tobacco control policies do not occur in isolation and plain packaging would be more effective if combined with media campaigns and fiscal policies.

Examples of experts’ reasoning in generating estimates:

Impact on adults

– For smoking prevalence to change substantially in two years, rather mega things have to happen. The proportion of smokers succeeding in stopping if trying is around 2½-3%, so even if every smoker tried, that’s only 3% of 21% change in prevalence. A half to a one percent change in prevalence is incredibly difficult to achieve [UK].

– The effect of plain packaging is likely to be quite small, as we found with the ban on smoking in public places. It feels like this is an important issue, but sometimes you just don’t know the level of impact this will have [UK].

– The extent of the effect depends on the number of younger smokers reflected in the current smoking prevalence rate. The effect will be a failure to replace older smokers with younger ones – recruitment will be affected with a failure to attract young smokers, whereas for well-established smokers there’ll be less impact [Australasia].

Impact on children

– From just looking at the evidence – attractiveness of product, smoking-related beliefs and behaviours – most indicate greater effects in children, so I’m more certain plain packaging would have an effect here. If we look at adult smokers – addicted smokers – they’re likely to be less interested in the packaging, and more on getting their hit, so are more likely to carry on smoking. Plain packaging is likely to impact on not starting smoking, to have more of an effect on uptake and experimentation [UK].

– There will be a reduction – plain packaging will break down brand awareness. I’m not convinced that there’ll be much impact in 2 years – the greater impact will be after this, with the cohort of children who have been less exposed. After two years the gains are considerable, but there’ll be much greater gains later on and then you’ll see the real benefit [North America].

General comments on impact

– I see plain packaging as part of an overall strategy, and the sum of the parts may be greater than the individual contribution, as they are all moving together, and it makes it hard to disaggregate the effects of one part [UK].

– As is already happening following the ad ban, there is a shift to economy, ultra-low price tobacco, with premium brands losing market share. The market is more driven by price than it used to be, and this would be reinforced by plain packaging. It would be difficult to invest in branding, and there would be a proliferation of low cost brands. Pricing could defeat the aims of the policy, so fiscal policy is needed to make sure this doesn’t occur [North America].

## Discussion

In the absence of direct evidence for the impact of plain packaging of tobacco products, this sample of tobacco control experts believe such a policy is likely to lead to a decline in smoking prevalence, and in particular, to a decline in the numbers of children trying smoking, two years after the introduction of plain packaging. No experts felt that the most likely outcome would be an increase in rates for either adults or children, and in each case the median lower estimate of the change was 0%, indicating a strong consensus that plain packaging would not increase consumption, assuming all else stayed equal. These findings were supported by the reasoning provided by experts, the majority of whom stated that they would expect a larger impact on uptake and therefore on the numbers of children smoking. The results are in line with the consistent findings from studies in this area that plain packaging is likely to impact on smoking prevalence [14].

The results provide the best guess estimates of a sample of international experts, along with a quantification of their uncertainty regarding the impact of plain packaging, using a method that provides independently generated estimates, in contrast with consensus development methods such as the Delphi method [30]. While previous studies have asked the general population about the likely impact of plain packaging on smoking [8,31,32], this study collates the views of tobacco control experts, who are able to put this into the perspective of other tobacco control measures implemented previously. Experts noted that previous policies tended to lead to reductions in adult smoking prevalence in the region of a half to one percentage point per annum, with bans on smoking in public places, advertising bans, price increases and educational campaigns used as reference points to place the impact of plain packaging into the tobacco control context. Even so, many experts mentioned that they were uncomfortable with providing a precise estimate for the impact, given the lack of direct evidence, and a few declined to give numerical estimates on this basis. This uncertainty is reflected in the heterogeneity between responses within regions. The study method means that the results were based on subjective judgements, albeit those of highly-informed individuals. Future research could compare these results with the actual impact of plain packaging, to inform understanding of the validity of experts’ estimates by looking at the accuracy of these predictions.

One potential limitation of the current study is the different response rates for the regions (high in the UK and Australasia but considerably lower for North America), although any resulting bias is unlikely given that no differences in estimates were found between regions. (The lower response rate from North America is perhaps due to the majority of invitees being from the US (with a minority from Canada), where plain packaging is unlikely to be implemented soon). This lack of discernible difference is particularly noteworthy given legal and political differences between these regions (with differences in key areas including the extent to which smoking is restricted in enclosed spaces, use of pack warnings and bans on tobacco advertising [33]). A further limitation of the current study is the sampling frame, which was solely on the basis of expertise in the area of tobacco control policies, leading to various degrees of familiarity with plain packaging policies and with the more economically-based arguments presented against the introduction of such policies. While all experts are likely to have knowledge of these issues (and all were given a recent systematic review), due to the general recruitment criteria used, we cannot determine whether responses differed by type of expertise. A more substantial concern regards the need to impose restrictions on estimates in the form of using a hypothetical scenario, i.e. all other factors remaining constant, which does not reflect reality, as noted by many participants. Several participants talked of the importance of tobacco control policies acting in concert, noting that if plain packaging were to be most effective, it should happen alongside media campaigns and taxation policies to ensure that the price of cigarettes is not driven down. In addition, only one time period (2 years post-policy introduction) was considered. Many participants felt that this was not sufficient time to see fully the impact of plain packaging in prevalence rates, suggesting a greater impact would be seen longer-term, as the impact on young people starting smoking fed through into adult smoking prevalence.

## Conclusions

In summary, while there remains considerable uncertainty about the likely impact of plain packaging of tobacco products given the policy has just been implemented for the first time, the views of experts in tobacco control are that such a policy will reduce smoking rates and that this will be greatest in children. None viewed an increase in smoking as the most likely outcome.

## Competing interests

The authors have no connections to the tobacco industry, nor any financial or non-financial competing interests that relate to the area of this study.

Participants

Australasia: Two have received funding for consultancy from pharmaceutical companies for smoking cessation; one has contracts with the New Zealand Ministry of Health to work on smoking cessation messages; one is on a Technical Advisory Committee advising the Commonwealth on design and implementation of plain packaging for tobacco products; two work for the Cancer Council Organisation (Australia); one is associated with Action on Smoking and Health (Australia).

UK: One has received funding from Action on Smoking and Health (ASH) UK for research on plain packaging; two are associated with ASH UK; one is associated with the Cancer Research UK Tobacco Advisory Group; two are associated with the Royal College of Physicians’ Tobacco Advisory Group; one is associated with the UK Centre for Tobacco Control Studies; six have undertaken research and consultancy for, and/or received honoraria for speaking at meetings for the manufacturers of smoking cessation medications/products; one has a share of a patent for a novel nicotine delivery device.

North America: one is serving as an expert witness for the Australian government on litigation concerning plain packaging of tobacco products; one has advised different government agencies with regard to plain packaging of tobacco products.

## Authors’ contributions

All authors were involved in study design, data interpretation and writing the report. RP collected the data and DS conducted the statistical analyses. All authors read and approved the final manuscript.

## Pre-publication history

The pre-publication history for this paper can be accessed here:

http://www.biomedcentral.com/1471-2458/13/18/prepub

## References

[B1] Baxter PJ Aspinall WP Neri A Zuccaro G Spence RJS Cioni R Woo G Emergency planning and mitigation at vesuvius: a new evidence-based approach J Volcanol Geotherm Res 2008 178 3 454 473 10.1016/j.jvolgeores.2008.08.015

[B2] Oppenheimer M O’Neill BC Webster M Agrawala S The limits of consensus Science 2007 317 5844 1505 1506 10.1126/science.1144831 17872430

[B3] Fayers PM Cuschieri A Fielding J Craven J Uscinska B Freedman LS Sample size calculation for clinical trials: the impact of clinician beliefs Br J Cancer 1999 82 1 213 219 10638992 10.1054/bjoc.1999.0902 PMC2363196

[B4] World Health Organisation WHO Framework Convention on Tobacco Control 2003 Geneva, Switzerland: WHO

[B5] Australian Government Tobacco Plain Packaging Act 2011: An Act to discourage the use of tobacco products, and for related purposes 2011 Canberra: Australian Government

[B6] Australian Government Tobacco Plain Packaging Amendment Regulation 2012 (No 1) 2012 Canberra: Australian Government

[B7] UK Department of Health Healthy lives, healthy people: a tobacco control plan for England 2011 London: UK Government

[B8] Goldberg M Leifeld J Kindra G Madill-Marshall J Lefebvre J Martohardjona N Vredenberg H When Packages Can’t Speak: Possible impacts of plain and generic packaging of tobacco products 1995 Toronto, Canada: prepared for Health Canada

[B9] Hoek J Wong C Gendall P Louviere J Cong K Effects of dissuasive packaging on young adult smokers Tob Control 2011 20 3 183 188 10.1136/tc.2010.037861 20966135

[B10] Moodie C Mackintosh AM Hastings G Ford A Young adult smokers’ perceptions of plain packaging: a pilot naturalistic study Tob Control 2011 20 367 373 10.1136/tc.2011.042911 21752795

[B11] Thrasher JF Rousu MC Hammond D Navarro A Corrigan JR Estimating the impact of pictorial health warnings and “plain” cigarette packaging: evidence from experimental auctions among adult smokers in the United States Health Policy 2011 102 1 41 48 10.1016/j.healthpol.2011.06.003 21763026

[B12] Wakefield MA Germain D Durkin SJ How does increasingly plainer cigarette packaging influence adult smokers’ perceptions about brand image? An experimental study Tob Control 2008 17 6 416 421 10.1136/tc.2008.026732 18827035 PMC2590906

[B13] Freeman B Chapman S Rimmer M The case for the plain packaging of tobacco products Addiction 2008 103 4 580 590 10.1111/j.1360-0443.2008.02145.x 18339104

[B14] Moodie C Stead M Bauld L McNeill A Angus K Hinds K Kwan I Thomas J Hastings G O'Mara-Eves A Plain tobacco packaging: A systematic review 2012 London: Public Health Research Consortium

[B15] Snowdon C Plain Packaging: Commercial expression, anti-smoking extremism and the risks of hyper-regulation 2012 London, UK: Adam Smith Institute

[B16] Padilla J The impact of plain packaging of cigarettes in UK: a simulation exercise 2010 Brussels: LECG Consulting Belgium, prepared for Philip Morris International

[B17] Reed H Analysis and review of J. Padilla, “The impact of plain packaging of cigarettes in the UK: a simulation exercise” 2011 Colchester, UK: Landman Economics, prepared for Action on Smoking and Health

[B18] Gervais A O’Loughlin J Meshefedjian G Bancej C Tremblay M Milestones in the natural course of onset of cigarette use among adolescents Can Med Assoc J 2006 175 3 255 261 10.1503/cmaj.051235 16880445 PMC1513423

[B19] Hora SC Von Winterfeldt D Nuclear waste and future societies: a look into the deep future Technol Forecast Soc Chang 1997 56 2 155 170 10.1016/S0040-1625(97)00075-9

[B20] Clemen RT Winkler RL Limits for the Precision and Value of Information from Dependent Sources Oper Res 1985 33 2 427 442 10.1287/opre.33.2.427

[B21] Hora SC Probability Judgments for Continuous Quantities: Linear Combinations and Calibration Manag Sci 2004 50 5 597 604 10.1287/mnsc.1040.0205

[B22] SHELF: the Sheffield Elicitation Frameworkhttp://www.tonyohagan.co.uk/shelf/

[B23] Papathomas M Hocking RJ Bayesian Updating for Binary Variables: An Application in the UK Water Industry Journal of the Royal Statistical Society Series D (The Statistician) 2003 52 4 483 499 10.1046/j.0039-0526.2003.00451.x

[B24] Paddock SM Ebener P Subjective prior distributions for modeling longitudinal continuous outcomes with non-ignorable dropout Stat Med 2009 28 4 659 678 10.1002/sim.3484 19012279 PMC2871545

[B25] Australian Institute of Health and Welfare 2010 National Drug Strategy Household Survey report Drug statistics series 2011 Canberra: AIHW

[B26] Bridges S Gill V Omole T Sutton R Wright V Smoking, Drinking and Drug Use among Young People in England in 2010 2011 London: NHS Information Centre for Health and Social Care

[B27] Reid JL Hammond D Tobacco Use in Canada: Patterns and Trends, 2011 Edition 2011 Waterloo, ON: Propel Centre for Population Health Impact, University of Waterloo

[B28] Robinson S Harris H Smoking and drinking among adults, 2009: A report on the 2009 General Lifestyle Survey 2011 London, UK: The Office for National Statistics

[B29] Smith G White V Australian secondary school students’ use of tobacco, alcohol, and over-the-counter and illicit substances in 2008 2009 Melbourne, Australia: Centre for Behavioural Research in Cancer, Cancer Council Victoria

[B30] O’ Hagan A Buck CE Daneshkhah A Eiser JR Garthwaite PH Jenkinson DJ Oakley JE Rakow T Uncertain Judgements: Eliciting Experts’ Probabilities 2006 Chichester, UK: John Wiley & Sons

[B31] Donovan R Smokers’ and non-smokers’ reactions to standard packaging of cigarettes 1993 Perth, Australia: University of Western Australia

[B32] Centre for Health Promotion Effects of plain packaging on the image of tobacco products among youth 1993 Toronto: University of Toronto

[B33] World Health Organisation WHO Report on the Global Tobacco Epidemic, 2009: Implementing smoke-free environments 2009 Geneva, Switzerland: World Health Organisation

